# Commentary: Taking care of people experiencing homelessness: a community case study on the practice of the Volunteer Association “A doctor for you” in Brescia, Italy

**DOI:** 10.3389/fpubh.2025.1725760

**Published:** 2026-01-12

**Authors:** Fabio De Giorgio, Beatrice Benedetti, Luigi Carbone, Massimo Ralli

**Affiliations:** 1 Department of Healthcare Surveillance and Bioethics, Section of Legal Medicine, Catholic University of the Sacred Heart, Rome, Italy; 2 Ospedale Fatebenefratelli Isola Tiberina - Gemelli Isola, Rome, Italy; 3 Departmental Faculty of Medicine, International Medical University, UniCamillus, Rome, Italy

**Keywords:** people experiencing homelessness, homelessness, medical liability, malpractice, vulnerable population

## 1 Introduction

We read with great interest the article by Pasini et al. (1). We highly appreciated the model of care described in the study and fully agree on the need to enhance the engagement of people experiencing homelessness (PEH) in the use of healthcare services. We also value the methodological approach adopted by the authors, which focuses on a single health topic of interest—chronic liver disease in this case—investigated through both instrumental examinations and a socio-educational framework based on participant observation and interviews. This design, in our view, represents an innovative and adaptable model that could be extended to other health conditions of relevance among PEH. In addition, we share the authors' emphasis on strengthening the continuity of care through the consistent involvement of healthcare and support workers. However, we believe that an additional topic of considerable importance deserves further reflection within this context—namely, the correct framing of the healthcare professional's role when operating in settings dedicated to the care of socially vulnerable populations such as PEH. This aspect is crucial not only for ensuring ethical and effective care delivery but also for clarifying potential issues of professional accountability that may arise in such complex and atypical work environments.

## 2 Peculiarities of health services provided to people experiencing homelessness

The public health literature has consistently documented high mortality among PEH, as recently confirmed by Nilsson et al. (2). Life expectancy in this population is markedly reduced, largely due to the high prevalence of psychiatric disorders and chronic diseases associated with significant disability—conditions that would benefit from timely and tailored medical interventions. Current healthcare provisions for PEH include street medicine programs (3) and specialized outpatient clinics. While expanding these services is essential, the growing involvement in PEH care raises medico-legal concerns that are likely to intensify in the future. Previous studies have highlighted the occurrence of potentially inappropriate prescriptions of psychoactive medications among PEH, which may contribute to adverse or even fatal outcomes (4). The peculiarities of PEH—marked by poor treatment adherence (5), a high prevalence of psychiatric disorders, and elevated risk of self-harm through prescribed medications or concomitant illicit substance use (6)—further heighten this risk. Additional barriers include communication difficulties (7) and the frequent lack of a general practitioner able to provide continuity of care (8). Together, these factors expose physicians in this field to a higher risk of professional liability compared with other healthcare settings.

## 3 Discussion

While integrated socio-healthcare approaches for PEH provide clear benefits for both individuals and the broader community, it remains crucial to explicitly define the legal framework within which such care is delivered. The provision of healthcare in informal or non-traditional settings often takes place under conditions of limited resources, fragmented documentation, and unclear lines of institutional responsibility. In Italy, a recent draft law approved by the Council of Ministers on September 4, 2025^1^, proposes the decriminalization of medical liability, limiting criminal prosecution to cases of gross negligence. This reform represents a potentially transformative step in promoting a more balanced relationship between professional accountability and the protection of healthcare workers across all sectors—yet it may have a particularly significant impact in the provision of healthcare to disadvantaged populations such as PEH. We believe that such a reform could increase both the number of healthcare professionals involved and their retention within socio-healthcare programs, thereby enhancing one of the key elements of homeless care: sustained patient engagement and the establishment of trust between PEH and healthcare providers. However, the issue of healthcare for PEH must also be considered in conjunction with the broader determinants of health. Beyond strengthening and legally protecting healthcare workers, it is essential to implement comprehensive support interventions—including nutritional assistance, access to personal hygiene facilities, and the safety and adequacy of shelters (9).

The right to healthcare for PEH is in fact supported by both European Union legal principles (10) and the national laws of individual member states, though with notable differences across countries.

In Germany, for instance, healthcare for PEH is provided within the standard healthcare system; as a result, uninsured individuals—who represent the majority of PEH—are often treated only for emergency conditions (11). In Italy, the National Health Service guarantees universal healthcare coverage; nevertheless, the lack of a permanent address frequently prevents homeless individuals from registering with a general practitioner, thus hindering continuity of care. In this respect, Law No. 176/2024 is a relevant advancement, as it allows access to general practitioners and essential levels of care through registration in municipal population registries, even for those without a fixed domicile (12).

Additional legislative frameworks worthy of mention are those established in the United States—both at the federal and state level—based on the principle of volunteer immunity. Under these laws, both healthcare and non-healthcare volunteers are protected from civil liability provided that certain conditions are met, including adherence to safety standards and the absence of willful misconduct or gross negligence (13). Such measures could foster a more supportive environment for healthcare professionals—both volunteers and contracted staff—encouraging them to work with socially vulnerable populations, including PEH, migrants, and individuals in detention. Reducing the fear of legal consequences would not only increase participation in community-based health programs but also strengthen the continuity and quality of care for marginalized groups.

In conclusion, we believe that a dual approach—combining the decriminalization of healthcare workers from a legal standpoint with the facilitation of healthcare access for PEH as exemplified by the community-based model proposed by Pasini et al. (1) —represents a fundamental objective to be pursued universally.

## Footnotes

1
https://www.governo.it/it/articolo/comunicato-stampa-del-consiglio-dei-ministri-n-140/29646?
